# Oligometastatic non‐small cell lung cancer: Impact of local and contemporary systemic treatment approaches on clinical outcome

**DOI:** 10.1002/ijc.35199

**Published:** 2024-09-25

**Authors:** Marcel Wiesweg, Claudia Küter, Johannes Schnorbach, Julius Keyl, Martin Metzenmacher, Jelena Cvetkovic, Felix Carl Saalfeld, Franziska Glanemann, Wilfried Eberhardt, Filiz Oezkan, Dirk Theegarten, Albrecht Stenzinger, Kaid Darwiche, Dirk Koschel, Felix Herth, Servet Bölükbas, Hauke Winter, Fabian Weykamp, Martin Wermke, Martin Stuschke, Till Plönes, Michael Thomas, Martin Schuler, Petros Christopoulos

**Affiliations:** ^1^ Department of Medical Oncology, West German Cancer Center, University Hospital Essen University Duisburg‐Essen Essen Germany; ^2^ Division of Thoracic Oncology, West German Cancer Center, University Medicine Essen—Ruhrlandklinik University Duisburg‐Essen Essen Germany; ^3^ Department of Thoracic Oncology, Thoraxklinik Heidelberg University Hospital Heidelberg Germany; ^4^ Institute of Pathology, West German Cancer Center, University Hospital Essen University Duisburg‐Essen Essen Germany; ^5^ Institute for Artificial Intelligence in Medicine, University Hospital Essen University Duisburg‐Essen Essen Germany; ^6^ Clinic for Internal Medicine I University Hospital, Technische Universität Dresden Dresden Germany; ^7^ National Center for Tumor Diseases (NCT/UCC) Dresden, and German Cancer Research Center (DKFZ) Heidelberg Germany; ^8^ Department of Pulmonary Medicine, Section of Interventional Pneumology, West German Cancer Center, University Medicine Essen—Ruhrlandklinik University Duisburg‐Essen Essen Germany; ^9^ Institute of Pathology, University Hospital Heidelberg Heidelberg University Heidelberg Germany; ^10^ National Center for Tumor Diseases (NCT) NCT Heidelberg, a partnership between DKFZ and Heidelberg University Hospital Heidelberg Germany; ^11^ National Center for Tumor Diseases (NCT), NCT West Essen Germany; ^12^ Department of Pneumology, Fachkrankenhaus Coswig, Lung Center, Coswig and Division of Pneumology, Medical Department I University Hospital Carl Gustav Carus, Technische Universität Dresden Dresden Germany; ^13^ Department of Pneumology and Critical Care Medicine Heidelberg University Hospital, Heidelberg University Heidelberg Germany; ^14^ Department of Thoracic Surgery and Endoscopy, West German Cancer Center, University Medicine Essen—Ruhrlandklinik University Duisburg‐Essen Essen Germany; ^15^ Department of Thoracic Surgery Thoraxklinik at Heidelberg University Hospital, Heidelberg University Heidelberg Germany; ^16^ Department of Radiation Oncology, University Hospital Heidelberg Heidelberg University Heidelberg Germany; ^17^ Heidelberg Institute of Radiation Oncology (HIRO) Heidelberg Germany; ^18^ Clinical Cooperation Unit Radiation Oncology German Cancer Research Center (DKFZ) Heidelberg Germany; ^19^ Department of Radiotherapy, West German Cancer Center, University Hospital Essen University Duisburg‐Essen Essen Germany; ^20^ Department of Thoracic Surgery, Fachkrankenhaus Coswig, Lung Center, Coswig and Division of Thoracic Surgery, Department of Visceral, Thoracic and Vascular Surgery Faculty of Medicine and University Hospital Carl Gustav Carus, Technische Universität Dresden Dresden Germany; ^21^ Helmholtz‐Zentrum Dresden—Rossendorf (HZDR) Dresden Germany; ^22^ Translational Lung Research Center Heidelberg (TLRC‐H) Member of the German Center for Lung Research (DZL) Heidelberg Germany

**Keywords:** immunotherapy, locally ablative treatment, multimodal concepts, observational study, oligometastatic NSCLC

## Abstract

Oligometastatic (OMD) non‐small cell lung cancer (NSCLC) is a distinct but heterogeneous entity. Current guidelines recommend systemic therapy and consolidation with local ablative therapy (LAT). However, evidence regarding the optimal choice of multimodal treatment approaches is lacking, in particular with respect to the integration of immunotherapy. This real‐world study identified 218 patients with OMD NSCLC (2004–2023, prespecified criteria: ≤5 metastases in ≤2 organ systems) from three major German comprehensive cancer centers. Most patients had one (72.5%) or two (17.4%) metastatic lesions in a single (89.9%) organ system. Overall survival (OS) was significantly longer with a single metastatic lesion (HR 0.54, *p* = .003), and female gender (HR 0.4, *p* < .001). Median OS of the full cohort was 27.8 months, with 29% survival at 5 years. Patients who had completed LAT to all NSCLC sites, typically excluding patients with early progression, had a median OS of 34.4 months (37.7% 5‐year OS rate) with a median recurrence‐free survival (RFS) of 10.9 months (13.3% at 5 years). In those patients, systemic treatment as part of first‐line therapy was associated with doubling of RFS (12.3 vs. 6.4 months, *p* < .001). Despite limited follow‐up of patients receiving chemo‐immunotherapy (EU approval 2018/2019), RFS was greatly improved by adding checkpoint inhibitors to chemotherapy (HR 0.44, *p* = .008, 2‐year RFS 51.4% vs. 15.1%). In conclusion, patients with OMD NSCLC benefitted from multimodality approaches integrating systemic therapy and local ablation of all cancer sites. A substantial proportion of patients achieved extended OS, suggesting a potential for cure that can be further augmented with the addition of immunotherapy.

## Graphical abstract

What's new?

The current treatment recommendations for oligometastatic non‐small cell lung cancer are based on evidence gathered prior to the immunotherapy era. This multicenter cohort study of 218 patients over the 2004–2023 period provides updated evidence that may guide the choice of systemic therapy for patients with oligometastatic non‐small cell lung cancer. The findings provide evidence supporting the benefit of multimodality approaches integrating immunotherapy‐based systemic treatment and local ablation of all cancer sites. Treatment goals should be prolonged disease control, with long‐term survival achievable in a subset of patients.

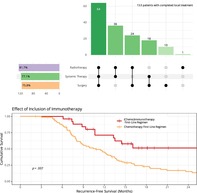

## 1 INTRODUCTION

Oligometastatic (OMD) non‐small cell lung cancer (NSCLC) is a distinct but heterogeneous entity. Definitions of synchronous oligometastatic disease^1^ applied in clinical trials^2^, ^3^ and reached by consensus boards^4^, ^5^ mostly agree on a maximum of 3–5 metastatic lesions in not more than 2–3 organ systems. Current guidelines recommend systemic treatment combined with locally ablative therapy (LAT) if technically feasible.^6^ This widely shared recommendation is based on a body of retrospective evidence,^7^, ^8^, ^9^, ^10^, ^11^, ^12^ a few smaller trials with restrictions in inclusion criteria or choice of LAT,^13^, ^14^, ^15^ and two randomized phase II trials that were both terminated early for interim efficacy analysis.^16^, ^17^ Randomized trials with different inclusion context (oligoprogression) or cross‐entity focus underlined the value of LAT in the context of oligometastases.^18^, ^19^, ^20^ Of note, all trials specific to synchronous oligometastatic NSCLC were conducted in the era before immunotherapy or chemo‐immunotherapy (chemo‐IO) became standard‐of‐care in the first‐line treatment of metastatic NSCLC. With current front‐line immunotherapies, 5‐year survival rates of up to 20%^21^, ^22^ can be achieved. Evidence on the role and value of available treatment modalities in OMD disease from the immunotherapy era is still limited: A single‐arm phase II trial showed favorable progression‐free survival (PFS) compared to historical controls in patients who received pembrolizumab after LAT. However, first‐line chemo‐IO was not administered.^23^ Two case series of patients with preoperative chemo‐immunotherapy detected high rates of pathological remissions in resected tumors and metastases,^24^, ^25^ consistent with findings from preoperative immunotherapy or chemo‐IO in early stage resectable NSCLC.^26^, ^27^, ^28^ A large Chinese single‐center series^29^ showed moderate benefit of additional LAT in patients receiving first‐line chemo‐IO. In a recent analysis applying systematic screening of lung cancer cases in the Netherlands, PFS was superior in 18 OMD NSCLC patients, the majority with PD‐L1‐high tumors, receiving chemo‐IO compared to a cohort of 50 patients treated with chemotherapy alone.^30^ Very recently, a large randomized trial including patients with either genuine synchronous OMD or induced oligopersistence after IO‐based treatments, and performing radiotherapy‐focused local ablation, reported no OS or PFS advantage.^31^

Still, evidence is lacking to guide the choice of optimal multimodal treatment approaches and systemic therapy regimens in the current treatment landscape. Such questions arise regularly in multidisciplinary lung cancer tumor boards, are controversially discussed^32^, ^33^ with variable outcomes across different lung cancer centers and tumor boards.

Against this background, we identified patients with OMD NSCLC treated at three large German comprehensive cancer centers over a decade and analyzed their clinical courses and outcome. The focus of this work was to capture the patient population as discussed in the regular lung cancer tumor boards, avoiding selection biases introduced by focusing on particular methods of LAT, or by restricting the analysis to those patients who qualify for LAT only after favorable outcome of initial therapy.

## 2 PATIENTS AND METHODS

### 2.1 Patient selection

Candidates with NSCLC potentially fulfilling OMD criteria and/or who had received multimodal treatment sequences for stage IV disease were identified (1) from clinical databases in which patients with OMD disease or OMD concept had been flagged prospectively, (2) by full‐text search in structured electronic health records for OMD‐specific terms in documents such as thoracic tumor board protocols, and (3) by searching in structured data for patients with metastatic lung cancer who had also undergone locally ablative procedures such as chemoradiotherapy or surgery. All candidates were manually verified to fulfill the criteria of synchronous, oligometastatic NSCLC,^1^ which were prespecified as ≤5 metastases in ≤2 organ systems,^5^ excluding primary tumor and mediastinal lymph nodes.

Locally ablative treatment sensu stricto (LAT), as used in further analysis, encompassed surgery or radiotherapy (other interventions, such as radio frequency ablation, may have been included but were not performed in our cohort) performed as part of the first‐line treatment sequence with the aim of local consolidation. This excluded palliative radiotherapy performed after disease progression.

### 2.2 Clinical data

Clinical data were retrieved from the electronic health records of participating centers. All analyses were performed on anonymized data sets. (Further details in the Supplementary Methods).

### 2.3 Clinical endpoints

We defined (1) overall survival (OS) as the time from initial diagnosis of NSCLC to death; (2) time to treatment failure (TTF) as time from initiation of a specific therapy to its discontinuation due to documented disease progression, switch to a new therapy line, decision to end cancer‐directed treatment for reasons other than durable tumor response (best supportive care), loss of contact, or death; and (3) recurrence‐free survival (RFS), only in patients who completed LAT, as the time from initiation of any therapy to first documented recurrence or disease progression, death, or any other TTF event. The latter implies that RFS as applied here is defined only for the subgroup of patients who completed LAT. As sequencing of local and systemic treatment varied widely, starting RFS from completion of local therapy would introduce a strong bias. We therefore defined RFS non‐traditionally to start from the initiation of any therapy, be it systemic treatment or any local procedure. TNM classification was based on the 8th edition of the UICC/IALSC staging system. (A complete set of analyzed variables is provided in the Supplementary Methods).

### 2.4 Multivariate analysis

Candidate prognostic features for univariate and multivariate analysis were age, sex, smoking history, ECOG status at diagnosis, histology (adenocarcinoma vs. others), N0‐1 versus N2‐3 status, N0 versus N1‐3 status, intra‐thoracic disease stage (stage I‐IIIC, disregarding the metastatic lesions), M1a, M1c versus M1a‐b, number of metastatic lesions (1–5), systemic therapy as part of first‐line treatment, first‐line systemic therapy containing immunotherapy, PD‐L1 expression on tumor cells, high PD‐L1 expression on tumor cells, TP53 mutation, and presence of a targetable alteration. For multivariate analyses, potential prognostic factors were filtered liberally for significance in univariate analysis (*p* < .2) to enter the multivariate regression. If multiple highly correlated features represented the same information (M status and number of metastases), only one entered the multivariate regression.

### 2.5 Data analysis

Data analysis and statistics were performed using R 4.2^34^ and the tidyverse.^35^ Plots, survival analyses (log‐rank tests, Cox proportional hazard model), and Kaplan–Meier plots were generated using the packages ggplot2 3.4,^36^ survival 3.5,^37^ survminer 0.4.9,^38^ and survivalAnalysis 0.4.0. Median follow‐up was assessed by the reverse Kaplan–Meier method. Confounder‐adjusted survival curves were generated using inverse probability of treatment weights (IPTW) weighted Kaplan–Meier curves based on a logistic regression propensity score,^39^, ^40^ as implemented by the “iptw_km” method in the adjustedCurves package.^41^ Associated *p*‐values were calculated by a modified version of the Pepe and Flemming test^42^ as implemented in the “adjusted_curve_test” method.

## 3 RESULTS

### 3.1 Patient population

We identified 218 patients diagnosed between 2004 and 2023 and treated at the three participating centers fulfilling the prespecified criteria of OMD NSCLC (≤5 metastases in ≤2 organ systems). Patient characteristics are given in Table 1. Patients were slightly younger (median age 60.6 years, 81% younger than 70) than unselected real‐world populations at our centers and mostly in favorable general condition (84.9% ECOG 0–1). PD‐L1 status, which was not part of the standard diagnostic workup prior to 2016 and therefore mostly not available for patients diagnosed in earlier years, was balanced (59% PD‐L1 positive [27.1% TPS ≥50%], 41% PD‐L1 negative, when documented). Fifteen patients had targetable genomic alterations (EGFR, 13; ROS1, 2). They were included in the analyses as applicable where they received OMD‐specific concepts rather than TKI‐based systemic therapy. Key results did not differ when excluding these patients.

**TABLE 1:** Patient characteristics (*n* = 218).

Sex	Female	102	46.8%
	Male	116	53.2%
Age at initial diagnosis	Median (min–max)	60.6 years (40.0–82.8)	
	Age below 70	176	80.7%
	Age 70 and above	42	19.3%
ECOG performance status	ECOG 0	110	50.5%
	ECOG 1	75	34.4%
	ECOG ≥2	13	6%
	Undocumented	20	9.2%
Time of diagnosis/eras of systemic treatment	Prior to 2010	4	1.8%
	2010–2013	20	9.2%
	2014–2017	60	27.5%
	Since 2018	134	61.5%
Tumor histology	Adenocarcinoma	179	82.1%
	Squamous	14	6.4%
	Adenosquamous	5	2.3%
	Large‐cell neuroendocrine	6	2.8%
	NOS and other	6	2.8%
	Undocumented	8	3.7
T stage	T1	41	18.8%
	T2	70	32.1%
	T3	53	24.3%
	T4	49	22.5%
	Tx/pT0/Undocumented	5	2.3%
N stage	N0	81	37.2%
	N1	26	11.9%
	N2	73	33.5%
	N3	35	16.1%
	Undocumented	3	1.4%
M stage (UICC 8)	M1a	16	7.3%
	M1b	144	66.1%
	M1c	58	26.6%
Affected organ systems	Metastases in one organ system	196	89.9%
	Metastases in two organ systems	22	10.1%
Targetable alterations	No targetable alteration detected or not tested	202	92.7%
	EGFR mutation	15	6.9%
	ROS1 fusion	1	0.4%
PD‐L1 TPS	PD‐L1 TPS <1%	59	41%
	PD‐L1 TPS 1%–49%	46	31.9%
	PD‐L1 TPS ≥50% (omitting 74 patients with undocumented PD‐L1)	39	27.1%

**FIGURE 1:**
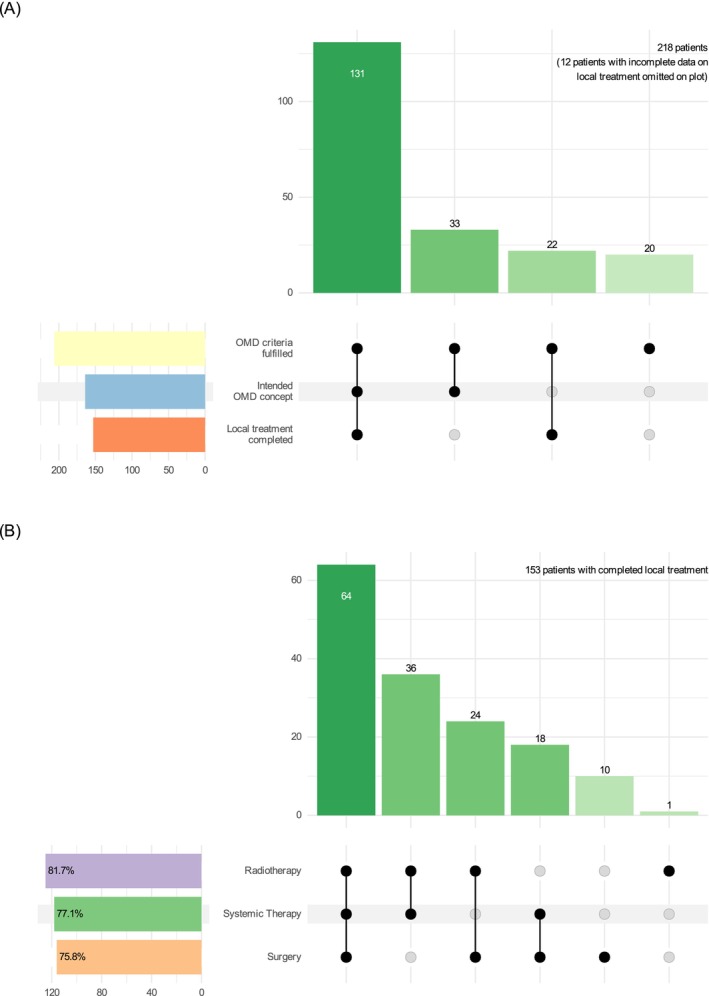
Visualizing subgroups and their intersections. (A) Prevalence of a documented OMD concept (a therapeutic concept involving locally ablative therapy of primary tumor and metastatic sites), and completion of locally ablative treatment. (B) Multimodality in patients who completed local treatment.

Of all 218 patients, 80.3% had a documented tumor board recommendation, or equivalent, of a therapeutic concept involving locally ablative therapy of primary tumor and metastatic sites (OMD concept group). 70.2% of patients, not an exclusive subgroup of those with an OMD concept, had documented completion of the intended LAT (Figure 1A). Median follow up of the full cohort was 32.1 months (48.5 months for patients who received chemotherapy‐based first‐line regimens, 18.1 months for patients who received [chemo]immunotherapy). Median OS of the full cohort was 27.8 months with a survival rate at 5 years of 29.0% and a median TTF of 9.5 months (Figure S1A,B for the LAT cohort).

### 3.2 Extent of OMD disease

**FIGURE 2:**
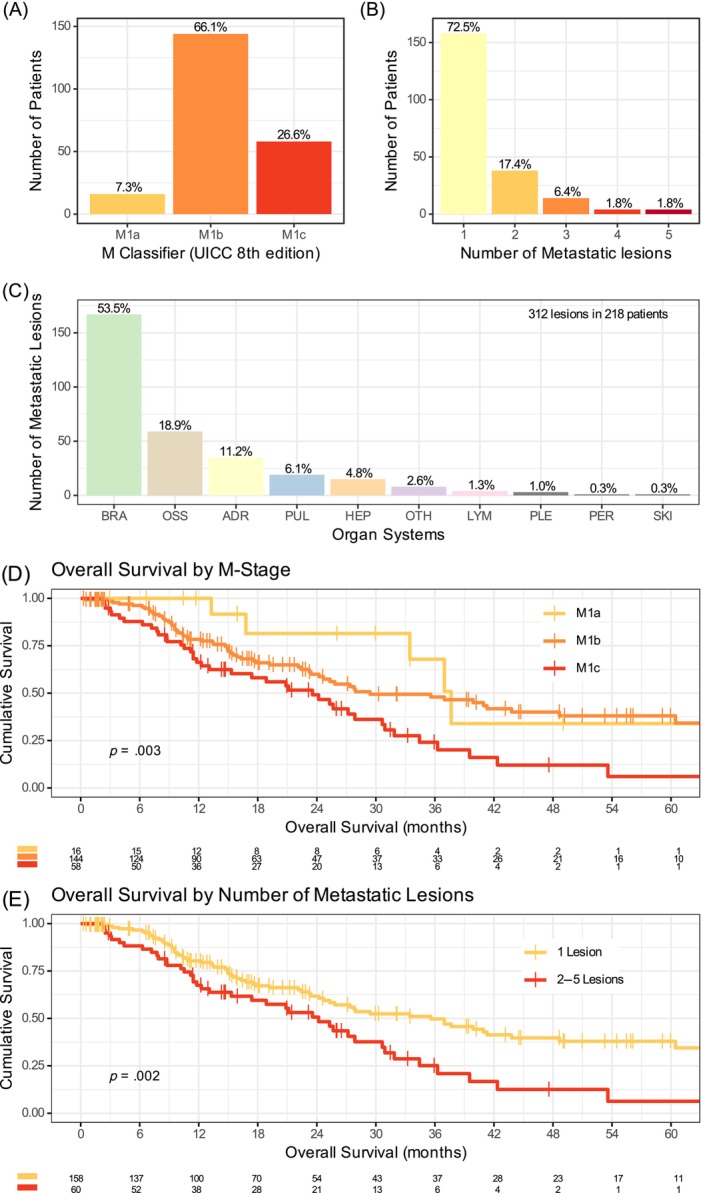
Extent of OMD disease and its prognostic impact. (A) M classifier, (B) Number of metastatic lesions per patient, and (C) Distribution of metastatic lesions over organ systems. Impact on overall survival of (D) the M classifier, and (E) the number of metastatic lesions.

Although the predefined criteria allowed up to five metastases, most patients had only one (72.5%) or two (17.4%) metastatic lesions in one (89.9%) organ system (Figure 2A). The 218 patients had a total of 312 metastases. Most frequently affected organ systems were brain (53.5%), bone (18.9%), adrenal gland (11.2%) and lung (6.1%, Figure 2B). This resulted in a large cohort of patients with true M1b disease per definition of the 8th edition of the UICC/IALSC staging system for lung cancer (66.1%, Figure 2C). Patients with M1a or M1b disease compared to M1c, and those with only one metastatic lesion compared to 2–5 metastatic lesions, had clearly superior OS (Figure 2D,E). Patients with metastases in lung, brain, bone or adrenal gland had superior OS compared to those with two affected organ systems or other metastatic locations (including liver, Figure S2).

### 3.3 Multimodality

**FIGURE 3:**
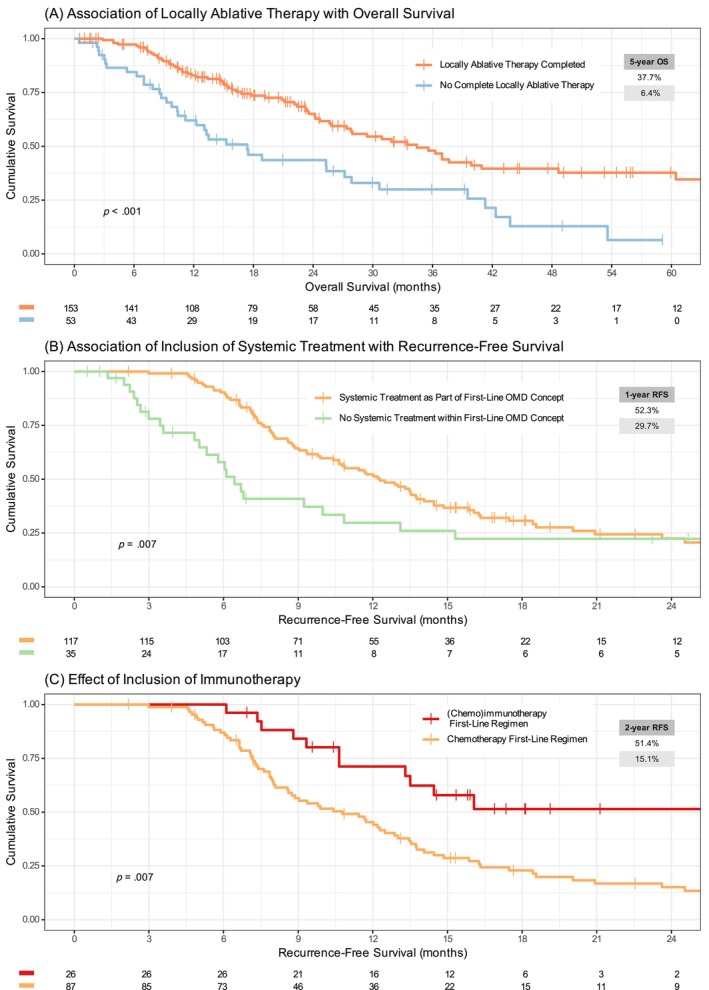
Association of multimodality treatment with survival endpoints. (A) Association of LAT with overall survival (*n* = 206, omitting 12 patients with insufficient documentation on completed LAT. Median OS 34.4 vs. 17.3 months, HR 0.46 [CI 0.31–0.69], *p* < .001, see discussion on confounding in main text). (B) Effect of inclusion of systemic treatment in the first‐line therapy on recurrence‐free survival (*n* = 152 patients who completed LAT and full information on systemic treatment. Median RFS 12.3 vs. 6.4 months, HR 0.55 [CI 0.35–0.85], *p* = .007). (C) Effect of inclusion of immunotherapy in the first‐line systemic treatment on recurrence‐free survival (*n* = 113 patients who completed LAT and received either (chemo‐)immunotherapy or chemotherapy as part of first‐line therapy. Median RFS 31.0 vs. 10.8 months, HR 0.44 [CI 0.24–0.81], *p* = .007).

A total of 153 patients (70.2% of full cohort, 74.9% of OMD concept group) completed LAT, consisting of radiotherapy (81.7%) and/or surgery (75.8%, Figure 1B). Patients whose LAT included surgery and those treated with radiotherapy only had similar outcomes (Figure S3). Completion of LAT was associated with superior OS (HR 0.46 [CI 0.31–0.69], *p* < .001, median OS 34.4 vs. 17.3 months), reaching a 5‐year OS rate of 37.7% (Figure 3A). Reasons for incomplete or no LAT were progressive disease (60.4%), no OMD concept offered at all (13.2%), LAT not offered after reevaluation for reasons of feasibility (9.4%), complete remission after systemic treatment (3.8%), or undocumented (13.2%). This shows that early disease progression is a strong confounder in the association of completed LAT with OS, with filtering by incomplete LAT effectively creating a subgroup selected by early disease progression and thus inferior OS. Patients who completed LAT had a median recurrence‐free survival of 10.9 months, with 13.3% of patients free of recurrence at 5 years.

Sequences of radiotherapy, surgery and systemic treatment were highly heterogeneous. In the LAT subgroup, 86 patients (56.2%) started treatment with surgery, 17 (11.1%) with radiotherapy and 50 (32.7%) received initially systemic treatment. 88 patients (57.5%) received both surgery and radiotherapy, 37 (24.2%) only radiotherapy and 28 (18.3%) were locally treated by surgery only.

Patients who did not receive systemic treatment in the first‐line setting (*n* = 46) were predominantly treated with initial surgery (80.4%). Possible reasons to evade systemic treatment, where documented, was missing recommendation in the “adjuvant” situation, patient refusal, postoperative complications, or delayed further contact with early recurrence.

### 3.4 Systemic treatment

In the full cohort, 172 patients (78.9%) received systemic treatment as part of first‐line treatment, prolonging TTF compared to those 45 patients who only received local therapy (median TTF, 11.0 vs. 3.9 months). The endpoint of recurrence‐free survival as defined for this analysis is restricted to patients who completed LAT. As shown above, this is a positively selected, but more homogeneous subgroup suitable for analysis of the effects of systemic treatment. 35 patients (22.9%) received LAT but no systemic treatment, 118 (77.1%) had documented systemic therapy. Systemic treatment significantly prolonged RFS (HR 0.55, *p* < .001, median RFS 12.3 vs. 6.4 months, Figure 3B). However, the RFS curves crossed at 2 years, and there was no significant OS benefit (HR 0.72, *p* = .24, median OS 35.5 vs. 27.3 months, Figure S4A,D). Chemo‐immunotherapy regimens became widely available per EMA approvals in 2018. Hence, the sample size and follow‐up of OMD NSCLC patients treated with chemo‐IO is still limited. 47 patients (21.6%) received first‐line chemo‐IO, 94 (43.1%) received immunotherapy during any treatment line. Baseline characteristics were balanced comparing patients receiving first‐line IO to those with no immunotherapy‐based first‐line treatment (Supplement Table). Among patients who completed LAT and received systemic therapy, 26 (22%) were treated with first‐line (chemo)immunotherapy, and 87 (73.7%) had received first‐line chemotherapy. Treatment with immunotherapy versus chemotherapy associated with a considerable improvement in RFS (HR 0.44, *p* < .008, median RFS 31.0 vs. 10.8 months, Figure 3C). At 2 years, 51.4% (CI 33.9%–78.0%) of patients who had completed LAT and received first‐line immunotherapy were free of recurrence, as compared to 15.1% (CI 8.7%–26.3%) who received first‐line chemotherapy. With only 5 events, OS data of the immunotherapy subgroup were immature and statistical significance could not be reached (HR 0.63, *p* = .331, median OS not reached vs. 34.4 months, Figure S4B,E). IPTW adjustment of survival curves with a propensity score based the significant prognostic baseline factors identified by multivariate analysis in this cohort (see next section), did not change these results (RFS, adjusted curves *p*‐value <.001, Figure S5; OS, *p* = .22, Figure S6). A benefit in OS, though, became apparent when comparing patients who received immunotherapy at least once during their treatment course, including second‐ and further‐line treatment, to those who never received IO (HR 0.66, *p* = .036, Figure S4C,F). The RFS benefit of immunotherapy was present in all PD‐L1 expression subgroups, but most pronounced in patients whose tumors expressed PD‐L1 on 1%–49% of tumor cells (Figure S7).

### 3.5 Prognostic baseline characteristics

**FIGURE 4:**
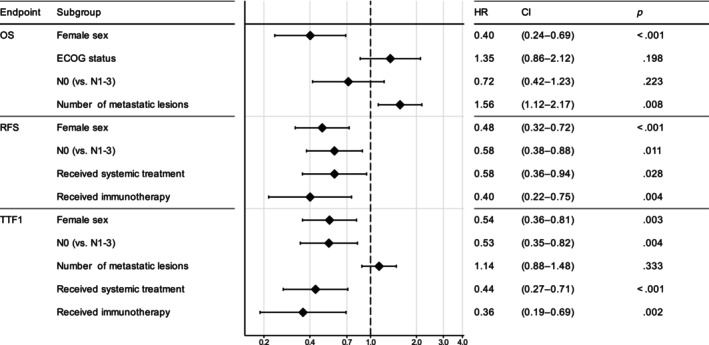
Multivariate analysis. Multivariate analysis of prognostic factors in patients who completed LAT for OS (*n* = 140 patients with complete data in analysis), RFS (*n* = 151) and TTF (*n* = 152).

We postulated that in addition to the already identified predictive information from metastatic disease spread and systemic treatment as outlined above, patient and tumor baseline characteristics as well as intra‐thoracic (T and N specifier, disregarding M) features were prognostic in OMD NSCLC. We restricted the analysis set to patients who completed LAT, representing a more homogenous population. Our dataset contained only few patients with liver metastases and was therefore not suited to validate the suggested adverse prognostic role of hepatic metastases.^43^ After liberal filtering for univariate significance (Figure S8, and see Methods for full list of candidate factors), multivariate analysis identified female sex (HR 0.40, *p* < .001) and the number of metastatic lesions (HR 1.56 per lesion, *p* = .008) as the only two significant prognostic factor for OS (Figure 4). For DFS and TTF, the same four significant prognosticators were identified: female sex, N0 status, systemic treatment as part of the first‐line therapy, and inclusion of immunotherapy in the first‐line regimen (Figure 4).

## 4 DISCUSSION

We present a multicentric cohort study of OMD NSCLC of sufficient sample size and quality of documentation to characterize real‐world outcomes and draw conclusions for future clinical practice.

We could identify a limited set of baseline prognostic factors, including female sex, N0 status, and the number of metastatic lesions. While the effect of nodal status and metastatic spread aligns with clinical expectation and previous reports,^44^, ^45^, ^46^ the very strong effect of female sex has not been noted to such extend in the cited works, which are based on data from the pre‐IO era. There is considerable debate and heterogeneous results regarding a potential gender bias particularly regarding the efficacy of immunotherapy.^47^, ^48^ Interestingly, women appeared to derive greater benefit from addition of chemotherapy to immunotherapy,^49^ which was also the prevalent regimen in our cohort.

Completion of LAT is usually a post hoc decision taken when information on response to systemic treatment is available. There is thus strong confounding by the occurrence of early disease progression when comparing groups with or without LAT in retrospective cohorts. In addition, a PFS benefit is highly likely when comparing LAT with no LAT as lesions that are able to grow are removed or radioablated, leaving the PFS endpoint to either failure of local control or development of new metastases.^33^ Retrospective case series such as many others and ours are therefore not suited to provide compelling evidence of the value of local treatment. Fortunately, data from a randomized, prospective trial is available showing an OS benefit of LAT^16^ in the pre‐IO era, establishing LAT as standard of care whenever feasible.^5^

OS of our full cohort (29% 5‐year OS), driven by the subgroup of patients having completed LAT (37.7% 5‐year OS), compared favorably to historical comparisons such as pivotal trials of chemo‐immunotherapy (18%–19% 5‐year OS),^21^, ^22^ even though many patients in our cohort did not receive immunotherapy. A similar favorable prognostic impact has also been described for oligoprogression under immunotherapy.^50^ At the same time, true long‐term freedom from recurrence, thus potential cure, was rare. Prolonged OS despite frequent recurrence may indicate specific biological disease features initially presenting as OMD with regard to susceptibility to further local or systemic therapy. Inclusion of systemic treatment led to a highly relevant advantage concerning the time to first progression or recurrence. We could not show an OS advantage in the analysis of systemic first‐line treatment, with data from the immunotherapy cohort still immature. Given a dramatic RFS advantage of the immunotherapy subcohort, and an OS advantage when receiving immunotherapy at least once during any line of treatment, an OS benefit in those patients receiving first‐line immunotherapy though appears likely.

As clear guidelines are lacking and new regimens became available, we observed a variety of concepts including initial systemic treatment, up‐front LAT followed by systemic treatment or LAT only as well as different systemic regimens. In contrast to the decision for LAT, the decision for and the choice of systemic treatment is an early, ex ante decision. Our data clearly supports the use of the most effective treatment regimen available, chemo‐immunotherapy. This is in line with recent data from a regional intention‐to‐treat analysis from the Netherlands,^30^ highlighting excellent PFS in a population of OMD NSCLC enriched with PD‐L1 high tumors. We learned from trials the neoadjuvant setting^26^, ^27^, ^28^ and case series in OMD NSCLC^24^, ^25^ that chemo‐IO is able to induce pathological complete responses in a relevant fraction of patients. Forgoing LAT due to complete remission under systemic treatment is a possible strategy that has been underrepresented in our cohort, but deserves further notice when designing OMD treatment strategies.

Observing a very favorable long‐term survival in a cohort of patients treated with LAT supports further pursuing this strategy whenever feasible. Still, toxicity and burden of treatment are relevant issues in a patient‐centric, individualized approach. Additional baseline risk factors affirmed in our cohort, such as sex, lymph node status and number of metastatic lesions, as well as treatment outcome after induction systemic therapy can help to reach a common decision on locally ablative procedures with the patient.

Possible limitations of our study are the consequence of its retrospective design: Aspects of the applied search methodology, such as requirement for electronic health records or prospective database documentation, yielded a patient population that was biased toward diagnosis in recent years. The majority of patients would have had access to immunotherapy (second‐line approval in 2015), and 60% of patients were diagnosed in the chemo‐immunotherapy era. All three centers feature a high caseload of primary lung cancer diagnosis, limiting the effect of center referral bias. Still, the applied search strategy did not include a screen of all primary diagnoses of stage IV NSCLC (in the three centers, approximately 9500 patients in the last decade) due to resource constraints and may thus miss cases fulfilling the inclusion criteria, but never flagged or identified as such.

The search strategy may also have excluded patients with “borderline” OMD with 3–5 metastases or a not clearly diagnosed number of lesions who were not recognized as having OMD. Still, the focus of our cohort on patients with 1–2 metastases reflects current guideline recommendations^5^ and clinical practice, and is further supported by the clearly inferior prognosis of patients with M1c disease or ≥2 metastases evident from our data.

In summary, our real‐world analysis supports multimodal OMD concepts including the most effective, immunotherapy‐based systemic treatment, and LAT of local tumor and metastatic lesions after careful consideration in an individualized treatment concept. Treatment goal should be prolonged disease control, with long‐term survival achievable in a subset of patients.

## AUTHOR CONTRIBUTIONS

**Marcel Wiesweg:** Conceptualization; data curation; formal analysis; investigation; methodology; project administration; resources; software; supervision; validation; visualization; writing – original draft; writing – review and editing. **Claudia Küter:** Data curation; investigation; methodology; resources; software; visualization; writing – original draft; writing – review and editing. **Johannes Schnorbach:** Investigation; resources; writing – review and editing. **Julius Keyl:** Investigation; resources; writing – review and editing. **Martin Metzenmacher:** Investigation; resources; writing – review and editing. **Jelena Cvetkovic:** Investigation; resources; writing – review and editing. **Franziska Glanemann:** Investigation; resources; writing – review and editing. **Felix Carl Saalfeld:** Investigation; resources; writing – review and editing. **Wilfried Eberhardt:** Investigation; resources; writing – review and editing. **Filiz Oezkan:** Investigation; resources; writing – review and editing. **Dirk Theegarten:** Investigation; resources; writing – review and editing. **Albrecht Stenzinger:** Investigation; resources; writing – review and editing. **Kaid Darwiche:** Investigation; resources; writing – review and editing. **Dirk Koschel:** Investigation; resources; writing – review and editing. **Felix Herth:** Investigation; resources; writing – review and editing. **Servet Bölükbas:** Investigation; resources; writing – review and editing. **Hauke Winter:** Investigation; resources; writing – review and editing. **Fabian Weykamp:** Investigation; resources; writing – review and editing. **Martin Wermke:** Investigation; resources; writing – review and editing. **Martin Stuschke:** Investigation; resources; writing – review and editing. **Till Plönes:** Investigation; resources; writing – review and editing. **Michael Thomas:** Conceptualization; formal analysis; investigation; project administration; resources; supervision; writing – original draft; writing – review and editing. **Martin Schuler:** Conceptualization; formal analysis; investigation; project administration; resources; supervision; writing – original draft; writing – review and editing. **Petros Christopoulos:** Conceptualization; formal analysis; investigation; project administration; resources; supervision; validation; writing – original draft; writing – review and editing.

## CONFLICT OF INTEREST STATEMENT

M. Wiesweg: Invited Speaker: Amgen, Roche, Takeda, GSK, AstraZeneca; Advisory Board: GSK, Novartis, Pfizer, Roche, Janssen, Daiichi Sankyo; Institutional research funding: Takeda, Bristol Myers Squibb; Travel support: Janssen, Amgen, Daiichi Sankyo. T. Plönes: Advisory Board: BMS; Invited Speaker: BMS, Roche, AstraZeneca. M. Metzenmacher: Advisory Board: AstraZeneca, Bristol Myers Squibb, MSD, Novartis, Novocure, Pfizer, Roche, Takeda. W.E. Eberhardt: Invited Speaker: AstraZeneca, Bristol Myers Squibb, MSD, Boehringer Ingelheim, Lilly, Amgen, Takeda, Pfizer, Novartis, Roche, Sanofi, AbbVie; Advisory Board: AstraZeneca, Roche, Bristol Myers Squibb, Bayer, Lilly, Boehringer Ingelheim, Takeda, Pfizer, MSD, Novartis, Sanofi, AbbVie, Amgen, Janssen; Institutional research funding: AstraZeneca, Lilly, Bristol Myers Squibb; Steering Committee Member: IASLC Staging Committee, M‐track. F.C. Saalfeld: Advisory Board: BMS, AstraZeneca, Janssen, Pfizer; Invited Speaker: AstraZeneca, Lilly, Janssen, Takeda, Pfizer, Novartis; Thieme, Deutsche Gesellschaft für Thoraxchirurgie, GWT‐TUD; Institutional research funding: Roche. M. Wermke: Advisory Board: BMS, Novartis, Lilly, Boehringer Ingelheim, ISA Pharmaceuticals, Immatics, Bayer, ImCheck Therapeutics; Invited Speaker: Lilly, Boehringer Ingelheim, SYNLAB, Janssen, Merck Serono, GWT TUD, Amgen, Novartis; Institutional research funding: Roche; Travel support: BMS, Pfizer, AstraZeneca, Amgen, GEMoaB, Sanofi/Aventis, Immatics, Merck Serono. A. Stenzinger: Advisory Board/Speaker's Bureau: Aignostics, Amgen, Astellas, Astra Zeneca, Bayer, BMS, Eli Lilly, Illumina, Incyte, Janssen, MSD, Novartis, Pfizer, Qlucore, Roche, Sanofi, Servier, Takeda, Thermo Fisher; Institutional research funding: Bayer, BMS, Chugai, Incyte. F. Weykamp: Invited Speaker: AstraZeneca, Varian Medical Systems, MSD; Travel support: Varian Medical Systems, Micropos Medical. M. Stuschke: Institutional research suppor: AstraZeneca. M. Thomas: Advisory Board: AstraZeneca, BeiGene, Bristol Myers Squibb, Boehringer Ingelheim, Celgene, Chugai, Daiichi Sankyo, GSK, Janssen, Lilly, Merck, MSD, Novartis, Pfizer, Roche, Sanofi, Takeda; Institutional research Funding: AstraZeneca, Bristol Myers Squibb, Merck, Roche, Takeda. M. Schuler: Invited Speaker: Amgen, Boehringer Ingelheim, Bristol Myers Squibb, Janssen, Novartis, Roche; Advisory Board: Amgen, AstraZeneca, Boehringer Ingelheim, Bristol Myers Squibb, GSK, Janssen, Merck Serono, Novartis, Roche, Sanofi, Takeda; Institutional research funding: AstraZeneca, Bristol Myers Squibb. P. Christopoulos: Advisory Board: AstraZeneca, Boehringer Ingelheim, Chugai, Pfizer, Novartis, MSD, Takeda, Roche, Daiichi Sankyo; Writing Engagement: Gilead; Invited Speaker: Thermo Fisher; Institutional research funding: AstraZeneca, Boehringer Ingelheim, Amgen, Novartis, Roche; Personal research funding: Takeda. All other authors have declared no conflicts of interest.

## ETHICS STATEMENT

As an observational study, data acquisition was approved by the institutional ethics committees of the participating Medical Faculties. Informed consent was waived.

## Supporting information


**Data S1.** Supporting information.

## Data Availability

The data that support the findings of this study are available from the corresponding author upon reasonable request.
