# Supplementary material for: Oligometastatic non‐small cell lung cancer: Impact of local and contemporary systemic treatment approaches on clinical outcome
Source: Int J Cancer. 2024 Sep 25;156(4):776–87. doi: 10.1002/ijc.35199 (PMC11661509; doi:10.1002/ijc.35199)
Supplement: Supplementary file 1 — Data S1. Supporting information. [file IJC-156-776-s001.pdf]

Supplement:
Oligometastatic non-small cell lung cancer: Impact of local and contemporary systemic treatment approaches on clinical outcome

Marcel Wiesweg, Claudia Küter, Johannes Schnorbach, Julius Keyl, Martin Metzenmacher, Jelena Cvetkovic, Felix Carl Saalfeld, Franziska Glanemann, Wilfried Eberhardt, Filiz Oezkan, Dirk Theegarten, Albrecht Stenzinger, Kaid Darwiche, Dirk Koschel, Felix Herth, Servet Bölükbas, Hauke Winter, Fabian Weykamp, Martin Wermke, Martin Stuschke, Till Plönes, Michael Thomas, Martin Schuler, Petros Christopoulos

Table of Contents

Supplemental Methods 2
Core variables 2
Statistics 2
Patient selection 3
Clinical Data 3
Supplemental Table 4
Supplemental Figures 5

Supplemental Methods

Core variables

Baseline characteristics

- Age at diagnosis
- Sex
- Smoking history
- TNM
- Histology
- ECOG status (initial diagnosis)
- Molecular pathology
    - KRAS
    - BRAF
    - EGFR
    - ALK
    - ROS1
    - RET
    - MET
    - STK11
    - KEAP1
    - Other relevant alteration
    - PD-L1
- Size of primary tumor
- Site and size of metastases

Disease and treatment characteristics

- Had documented OMD concept / date
- Has completed local treatment / date
- Treatment modalities (per treatment line)
- Systemic treatment regimens (per treatment line)
- Local treatments (per treatment line)
- Reason why locally ablative treatment not done

Outcome variables

- Overall survival
- Recurrence-free survival
- Time to treatment failure (per treatment line)
- Best reponse (real-world assessment in analogy to RECIST, per treatment line)

Statistics

As a retrospective study, all analyses should be formally considered descriptive and hypothesis generating. We aimed to avoid bias by including all identified patients, filtered only by baseline OMD disease characteristics (see main text). In particular, no patients were excluded due to early tumor progression or inadequate general condition. In consequence, there was no pre-planned sample size calculation, but the full available cohort was included. The pre-specified main aims of the study were analysis of OMD NSCLC concepts in relation to LAT against the background of current standard-of-

care systemic therapy. Therefore, we consider subgroup analysis by LAT, and by immunotherapy-based systemic treatment as pre-specified. Strong aspects of bias typically introduced by selection in retrospective analyses of LAT vs. no LAT became clear when we analysed reasons for missed LAT, and are described and discussed in the main text.

For identification of prognostic factors, we considered as candidate features, based on data availability, prevalence and prior knowledge: age, sex, smoking history, ECOG status at diagnosis, histology (adenocarcinoma vs. others), N0-1 vs. N2-3 status, N0 vs. N1-3 status, intra-thoracic disease stage (stage I-IIIC, disregarding the metastatic lesions), M1a, M1c vs. M1a-b, number of metastatic lesions (1-5), systemic therapy as part of first-line treatment, first-line systemic therapy containing immunotherapy, PD-L1 expression on tumor cells, high PD-L1 expression on tumor cells, TP53 mutation, and presence of a targetable alteration.
These variables entered univariate and possibly multivariate analysis as described in the main Methods.

Patient selection

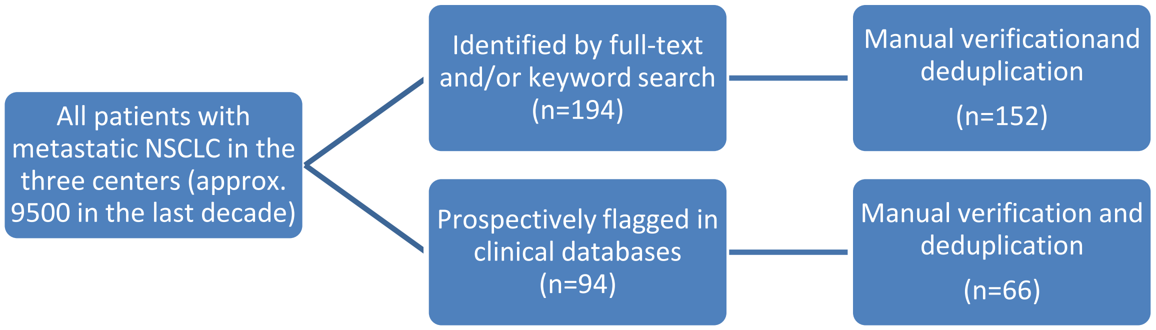

Patients were excluded only because they did not meet pre-specificed OMD disease criteria. There is no subgroup excluded from analysis in the full cohort for other reasons. Limitations of sensitivity in step 1 are discussed in the main text.

Clinical Data

Clinical data were retrieved from the electronic health records of participating centers. From raw clinical data, key events of oncological history were manually extracted, resulting in a high-level structured documentation of events (procedures, therapies, diagnostic findings, treatment decisions) in disease history (medial student / documentalist). This documentation was independently checked (physician: experienced thoracic oncologist) for plausibility, both on the patient level and by performing crosschecks on aggregate data for completeness and coherency. Data cut-off was April 2023, crosschecks and corrections for missing or incoherent data were complete in August 2023.

Supplemental Table

Patient Characteristics in cohorts defined by first-line regimen					
		Received first-line immunotherapy-based systemic therapy (n=47)		Received no immunotherapy as part of first-line treatment (n=171)	
**Sex**	Female<br>Male	22<br>25	46.8%<br>53.2%	80<br>91	46.8%<br>53.2%
**Age at Initial Diagnosis**	Median (Min – Max)	60.8 years (43.4 – 82.8)		60.2 years (40.0 – 82.6)	
	Age below 70<br>Age 70 and above	39<br>8	86%<br>17%	137<br>34	80.1%<br>19.9%
**ECOG Performance Status**	ECOG 0<br>ECOG 1<br>ECOG ≥ 2<br>Undocumented	24<br>19<br>3<br>1	51.1%<br>40.4%<br>6.4%<br>2.1%	86<br>56<br>10<br>19	50.3%<br>32.7%<br>5.8%<br>11.1%
**Time of diagnosis / Eras of Systemic Treatment**	Prior to 2010<br>2010 – 2013<br>2014 – 2017<br>Since 2018	 <br><br>47	 <br><br>100%	4<br>20<br>60<br>87	2.3%<br>11.7%<br>35.1%<br>50.9%
**Tumor Histology**	Adenocarcinoma<br>Squamous<br>Adenosquamous<br>Large-cell neuroendocrine<br>NOS and other<br>Undocumented	38<br>3<br>2<br>0<br>3<br>1	80.9%<br>6.4%<br>4.3%<br>0<br>6.4%<br>2.1%	141<br>11<br>3<br>3<br>6<br>7	82.5%<br>6.4%<br>1.8%<br>1.8%<br>3.5%<br>4.1%
**T Stage**	T1<br>T2<br>T3<br>T4<br>Tx/pT0/Undocumented	8<br>13<br>10<br>15<br>1	17.0%<br>27.7%<br>21.3%<br>31.9%<br>2.1%	33<br>57<br>43<br>34<br>4	19.3%<br>33.3%<br>25.1%<br>19.9%<br>2.6%
**N Stage**	N0<br>N1<br>N2<br>N3<br>Undocumented	20<br>5<br>12<br>10<br>-	42.6%<br>10.6%<br>25.5%<br>21.3%<br>-	61<br>21<br>61<br>25<br>3	35.7%<br>12.3%<br>35.7%<br>14.6%<br>1.8%
**M Stage (UICC 8)**	M1a<br>M1b<br>M1c	7<br>26<br>14	14.9%<br>55.3%<br>29.8%	9<br>118<br>44	5.3%<br>69.0%<br>25.7%
**Affected Organ Systems**	Metastases in one organ system<br>Metastases in two organ systems	41<br>6	87.2%<br>12.8%	155<br>16	90.6%<br>9.4%
**Targetable Alterations**	No targetable alteration detected or not tested	47	100%	155	90.6%
**PD-L1 TPS**	PD-L1 TPS < 1%<br>PD-L1 TPS 1 – 49%<br>PD-L1 TPS ≥ 50%<br>Undocumented (*omitted from percentage to allow comparison)	18<br>16<br>11<br>2	40.0%<br>35.6%<br>24.4%<br>*	41<br>30<br>28<br>72	41.4%<br>30.3%<br>28.3%<br>*
**First-line regimen**	Platinum/Taxane<br>Platinum/Pemetrexed<br>Targeted therapy<br>Platinum/Vinorelbine<br>Platinum/Etoposide<br>Platinum/other<br>No systemic first-line treatment<br>Platinum/Pem/Pembro<br>Carboplatin/Pacli/Pembro<br>Pembro mono	 <br><br><br><br><br><br>38<br>8<br>1	 <br><br><br><br><br><br>80.9%<br>17.0%<br>2.1%	60<br>20<br>6<br>27<br>7<br>5<br>46	35.1%<br>11.7%<br>3.5%<br>15.8%<br>4.1%<br>3.0%<br>26.9%

Supplemental Figures

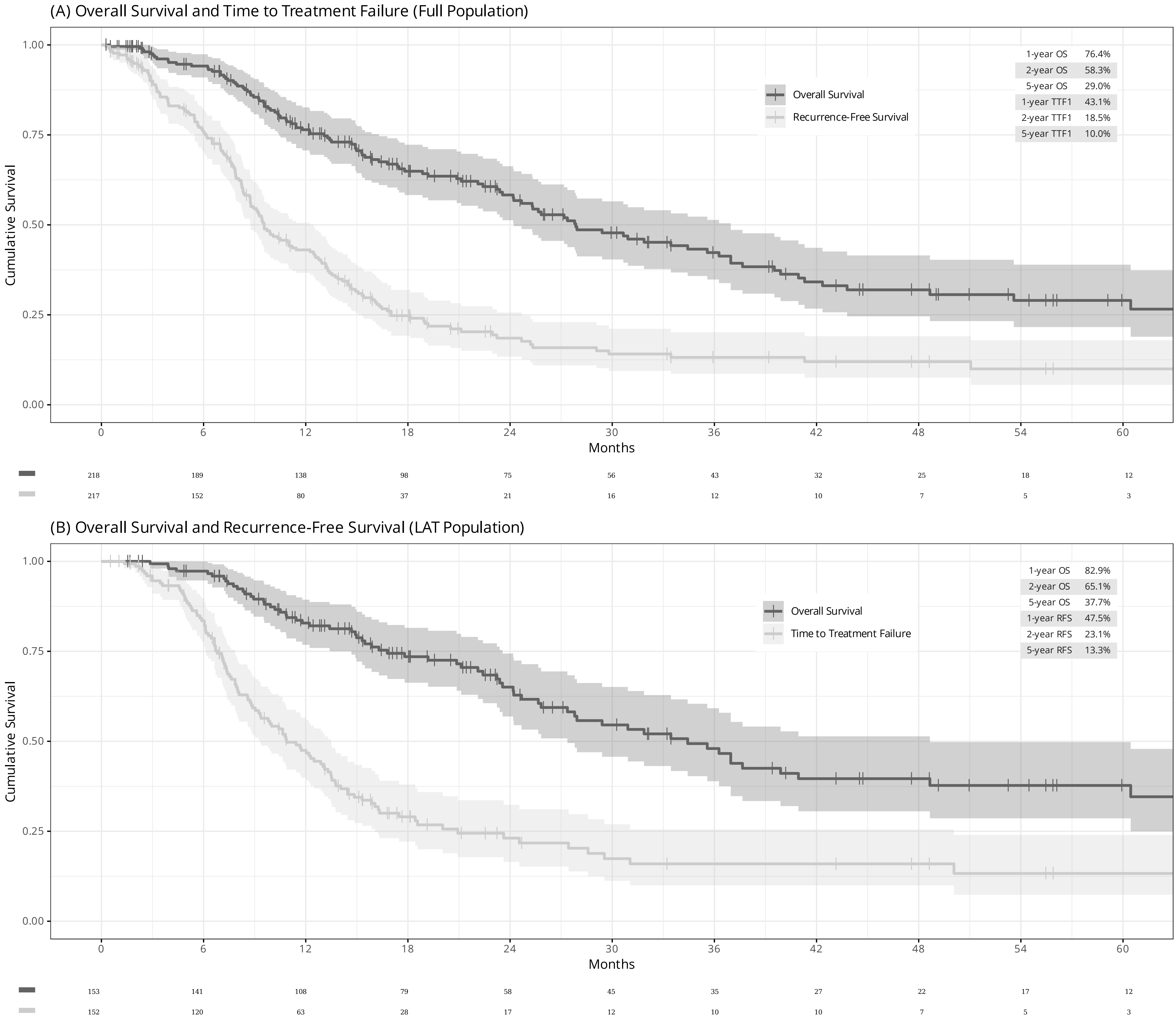

Figure S1: Overall survival, time to treatment failure and recurrence-free survival. Curves with different endpoints in one plot for illustration.
(A) Overall survival (n=218, median 27.8 months, CI 24.2 – 36.9) and time to treatment failure upon first-line therapy
(n=217, one patient with missing documentation of endpoint, median 9.5 months, CI 8.7 – 12.4) of the full cohort.
(B) Overall survival (n=153, median 34.4 months, CI 27.3 – 48.6) and recurrence-free survival
(n=152, one patient with missing documentation of endpoint, median 10.9 months, CI 9.3 – 13.4) of patients who completed LAT.

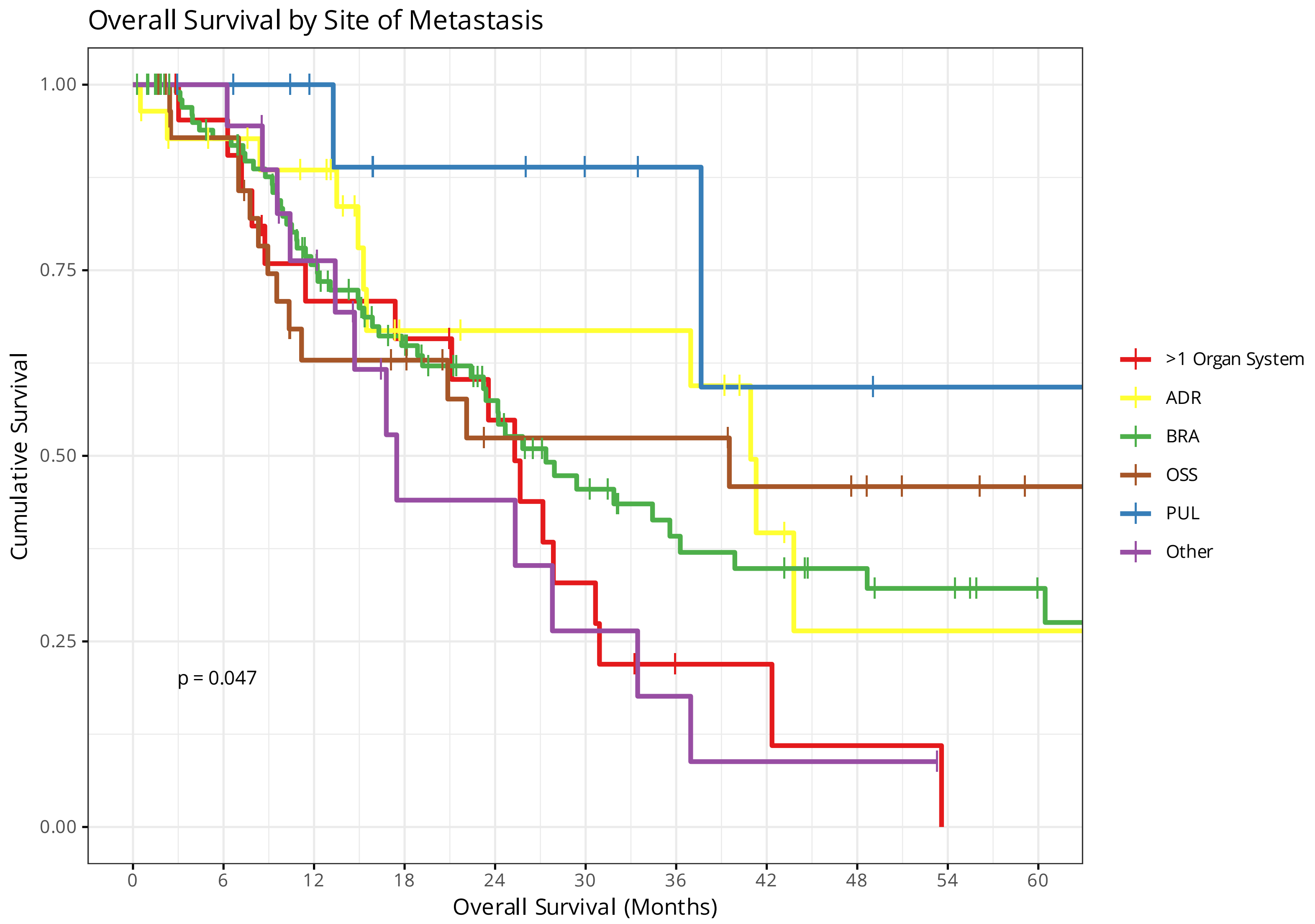

Figure S2: Extent of OMD disease and its prognostic impact: Site of metastases. “Other” groups HEP, OTH, LYM, PLE, PER and SKI (see also Fig. 2C).

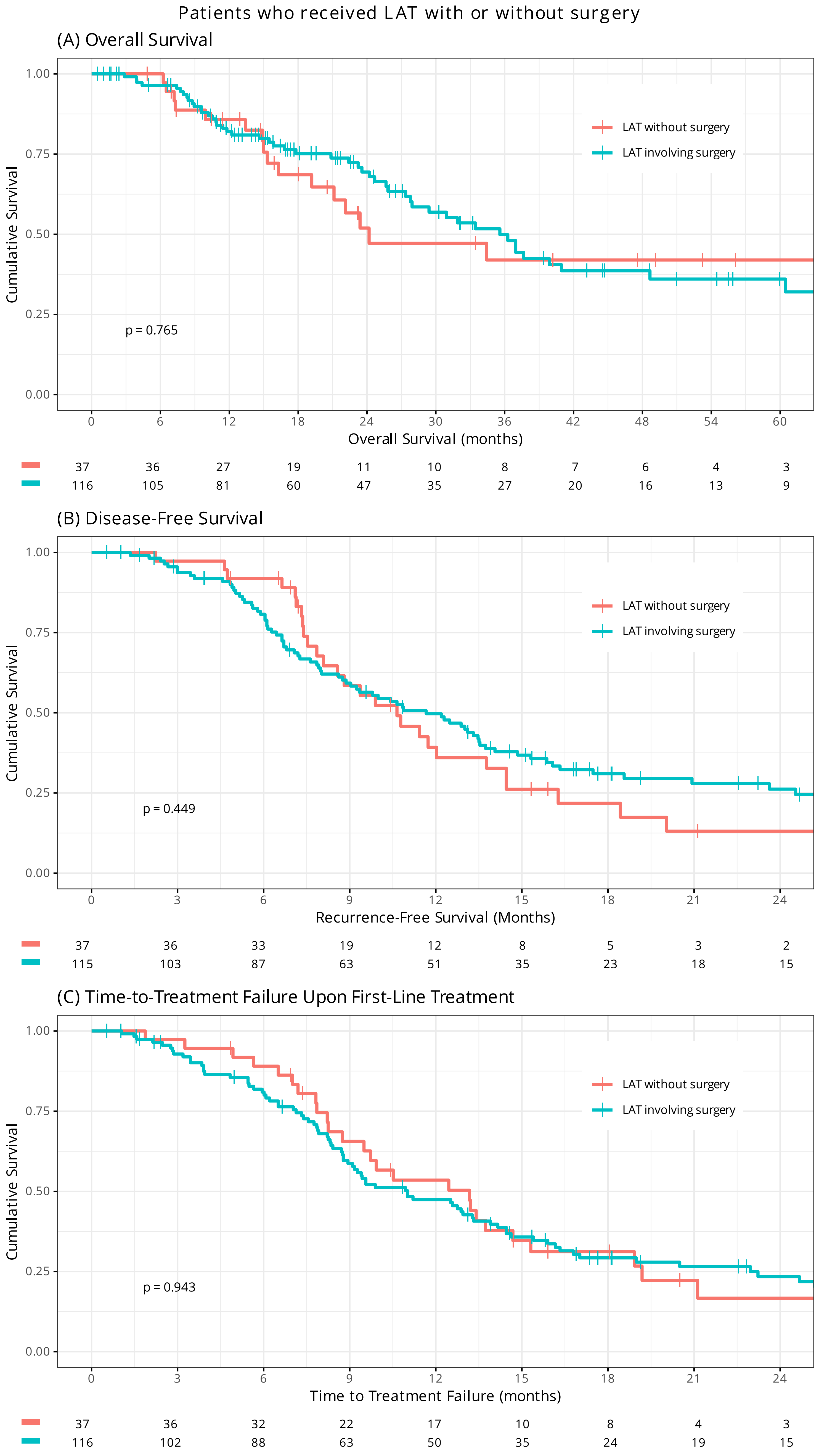

Figure S3: Association of the inclusion of surgical procedures into LAT with overall survival, recurrence-free survival and time to treatment failure.

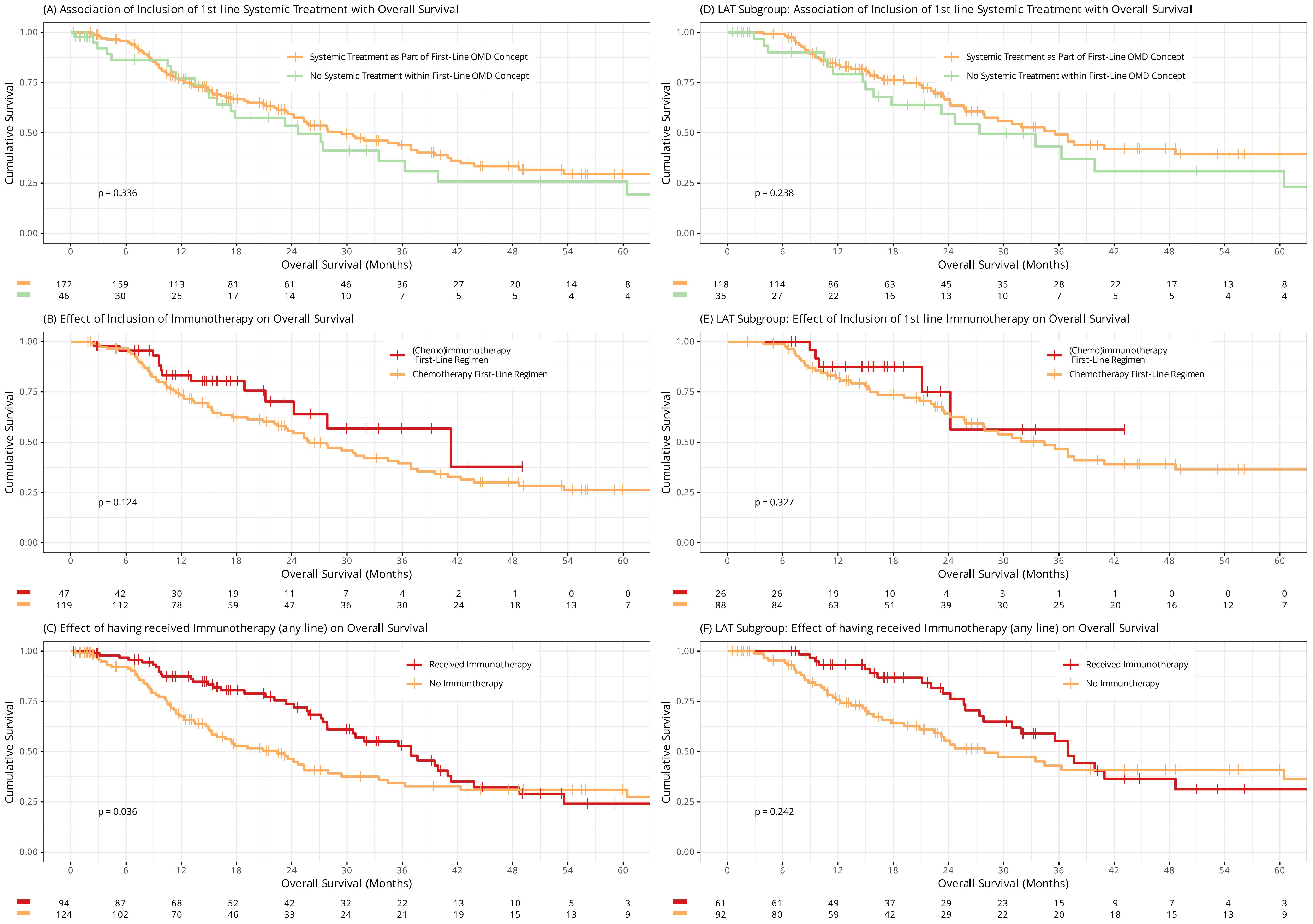

Figure S4: Effects of systemic treatment choices on overall survival in the full cohort (A, B, C) and the subgroup of patients who completed LAT (D, E, F), complementing Fig. 3B and 3C.
(A), (C): Effect of inclusion of systemic treatment in the first-line therapy.
(B), (D): Effect of inclusion of immunotherapy in the first-line systemic treatment.
(C), (F): Effect of receiving immunotherapy at least once, regardless of treatment line.

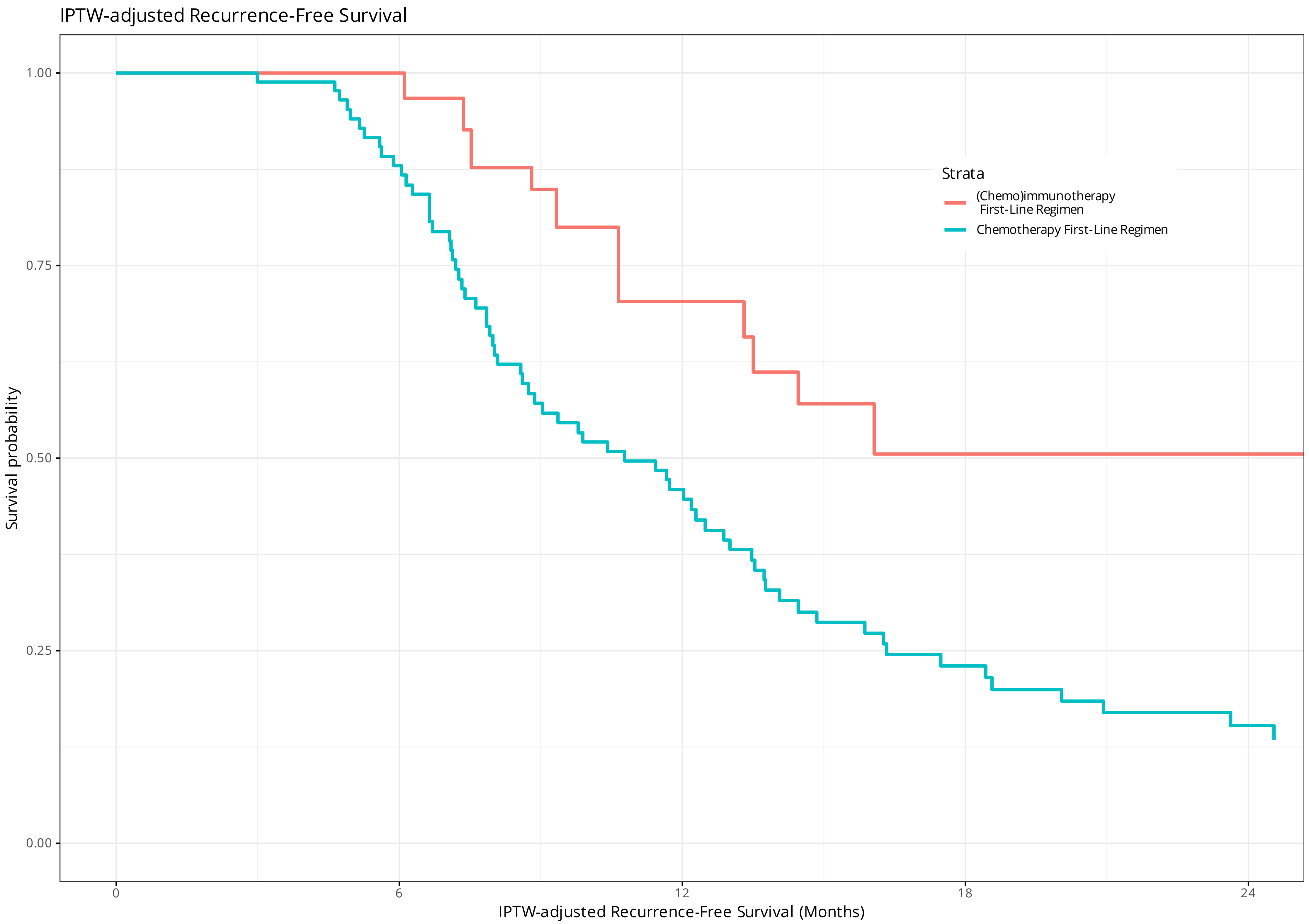

Figure S5: IPTW-weighted Kaplan-Meier curve based on a logistic regression propensity score
(see methods) of the effect of inclusion of immunotherapy in the first-line systemic treatment on recurrence-free survival. Associated p-value <0.001,
numbers at risk not defined.

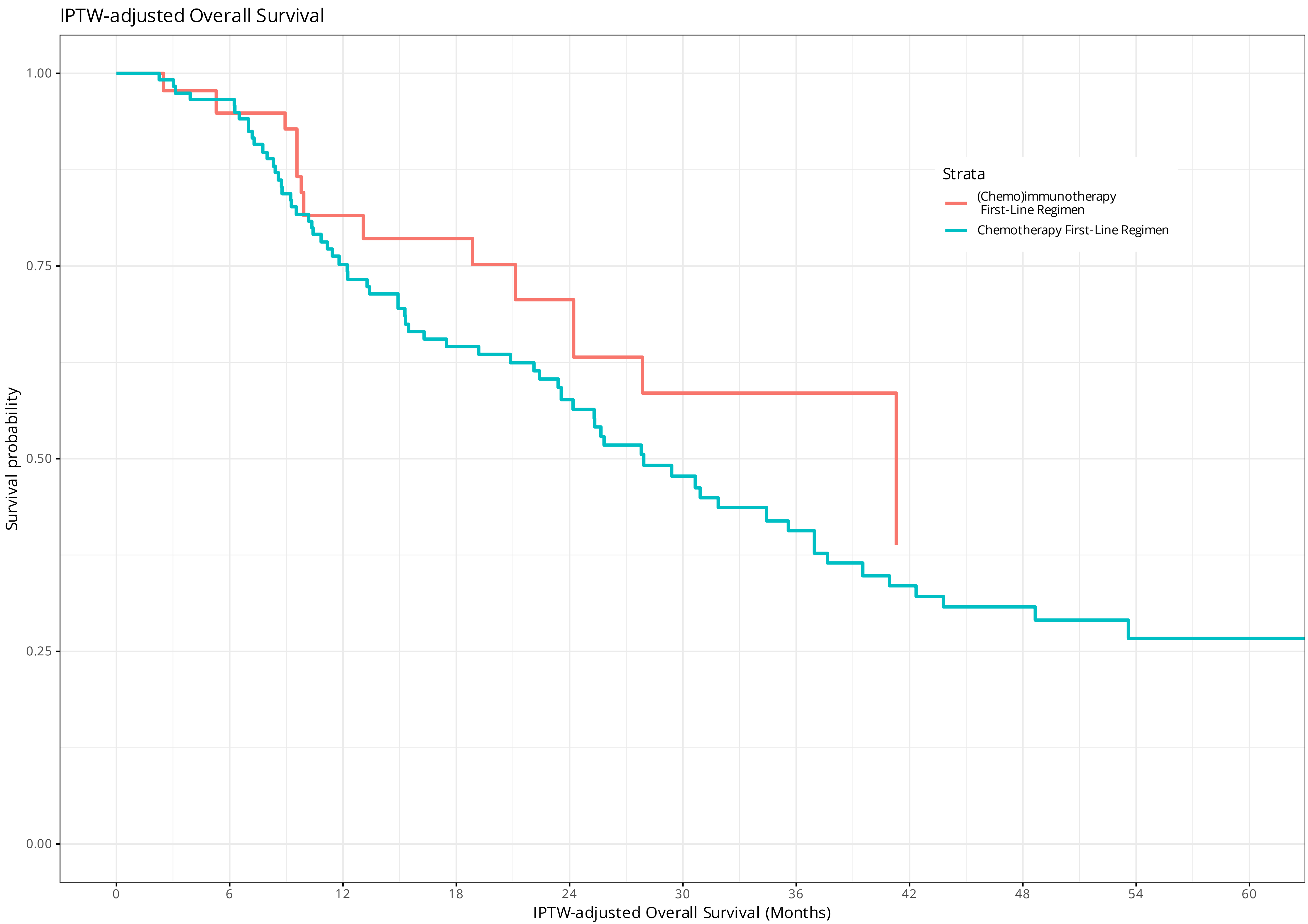

Figure S6: IPTW-weighted Kaplan-Meier curve based on a logistic regression propensity score (see methods) of the effect of inclusion of immunotherapy in the first-line systemic treatment on overall survival. Associated p-value 0.22, numbers at risk not defined.

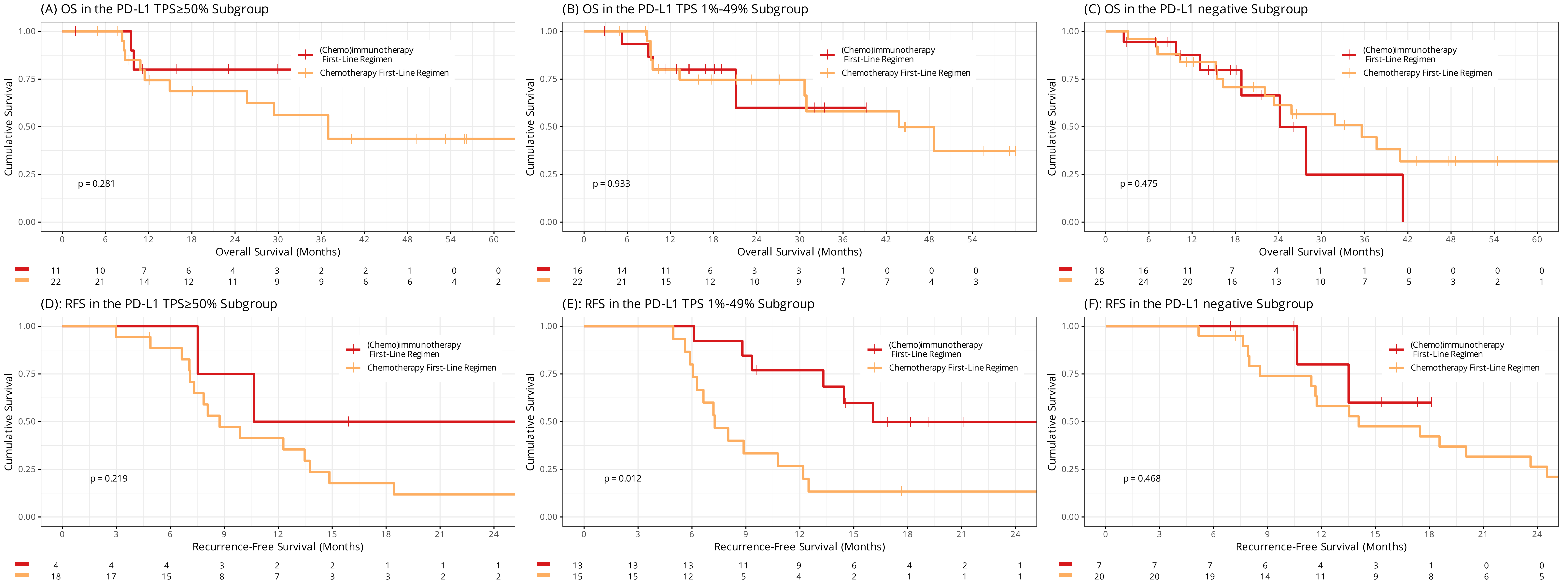

Figure S7: Stratification of the effect of inclusion of immunotherapy in the first-line systemic treatment on overall and recurrence-free survival
(43, 38 and 33 patients with available PD-L1 status, PD-L1 TPS negative, 1-49% and ≥50% respectively, who completed LAT and received either (chemo-)immunotherapy or chemotherapy as part of first-line therapy).

Endpoint	Subgroup	HR	CI	p	n
OS	age at dx	1.00	(0.97–1.03)	0.979	153
	Female sex	0.41	(0.25–0.68)	<0.001	153
	Smoking history	1.12	(0.60–2.07)	0.723	153
	Adenocarcinoma	1.02	(0.51–2.01)	0.962	147
	ECOG status	1.45	(0.93–2.26)	0.098	141
	N0-1 (vs. N2-3)	0.76	(0.47–1.24)	0.275	152
	N0 (vs. N1-3)	0.61	(0.36–1.02)	0.060	152
	Thoracic disease stage	1.12	(0.93–1.36)	0.239	148
	M1c (vs. M1a/b)	2.03	(1.18–3.49)	0.010	153
	M1a (vs. M1b/c)	0.79	(0.29–2.18)	0.650	153
	Number of metastatic lesions	1.78	(1.30–2.42)	<0.001	153
	Received systemic treatment	0.72	(0.42–1.24)	0.241	153
	Received immunotherapy	0.61	(0.24–1.53)	0.287	153
	PD-L1 high	0.93	(0.44–1.94)	0.843	97
	PD-L1 positive	0.96	(0.49–1.87)	0.897	97
	TP53 mutated	1.24	(0.63–2.42)	0.530	97
	Targetable alteration	0.71	(0.26–1.97)	0.513	153
RFS	age at dx	0.99	(0.97–1.01)	0.396	152
	Female sex	0.56	(0.38–0.82)	0.003	152
	Smoking history	1.31	(0.78–2.21)	0.305	152
	Adenocarcinoma	1.15	(0.66–2.03)	0.618	146
	ECOG status	0.95	(0.65–1.38)	0.774	140
	N0-1 (vs. N2-3)	0.94	(0.64–1.37)	0.731	151
	N0 (vs. N1-3)	0.75	(0.50–1.10)	0.142	151
	Thoracic disease stage	1.03	(0.89–1.19)	0.676	147
	M1c (vs. M1a/b)	1.07	(0.68–1.70)	0.766	152
	M1a (vs. M1b/c)	0.57	(0.25–1.29)	0.177	152
	Number of metastatic lesions	1.03	(0.76–1.41)	0.829	152
	Received systemic treatment	0.55	(0.35–0.85)	0.008	152
	Received immunotherapy	0.43	(0.24–0.78)	0.006	152
	PD-L1 high	1.38	(0.83–2.30)	0.213	97
	PD-L1 positive	1.36	(0.84–2.20)	0.208	97
	TP53 mutated	1.18	(0.73–1.90)	0.512	97
	Targetable alteration	0.65	(0.29–1.49)	0.314	152
TTF1	age at dx	0.99	(0.97–1.01)	0.315	153
	Female sex	0.56	(0.38–0.81)	0.003	153
	Smoking history	1.21	(0.74–1.98)	0.454	153
	Adenocarcinoma	1.06	(0.61–1.83)	0.830	147
	ECOG status	0.99	(0.69–1.42)	0.949	141
	N0-1 (vs. N2-3)	0.95	(0.65–1.39)	0.799	152
	N0 (vs. N1-3)	0.73	(0.50–1.08)	0.112	152
	Thoracic disease stage	1.03	(0.89–1.18)	0.718	148
	M1c (vs. M1a/b)	1.31	(0.85–2.04)	0.224	153
	M1a (vs. M1b/c)	0.51	(0.22–1.17)	0.112	153
	Number of metastatic lesions	1.21	(0.93–1.58)	0.158	153
	Received systemic treatment	0.42	(0.27–0.64)	<0.001	153
	Received immunotherapy	0.37	(0.20–0.69)	0.002	153
	PD-L1 high	1.07	(0.64–1.79)	0.801	97
	PD-L1 positive	1.08	(0.67–1.75)	0.739	97
	TP53 mutated	0.94	(0.59–1.51)	0.809	97
	Targetable alteration	0.87	(0.42–1.79)	0.708	153

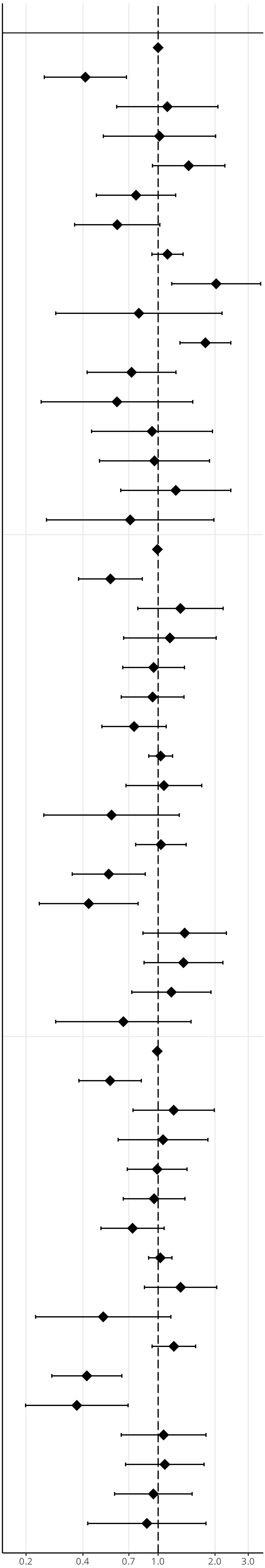

Figure S8: Univariate analysis of prognostic factors in patients who completed LAT for OS, RFS and TTF.
